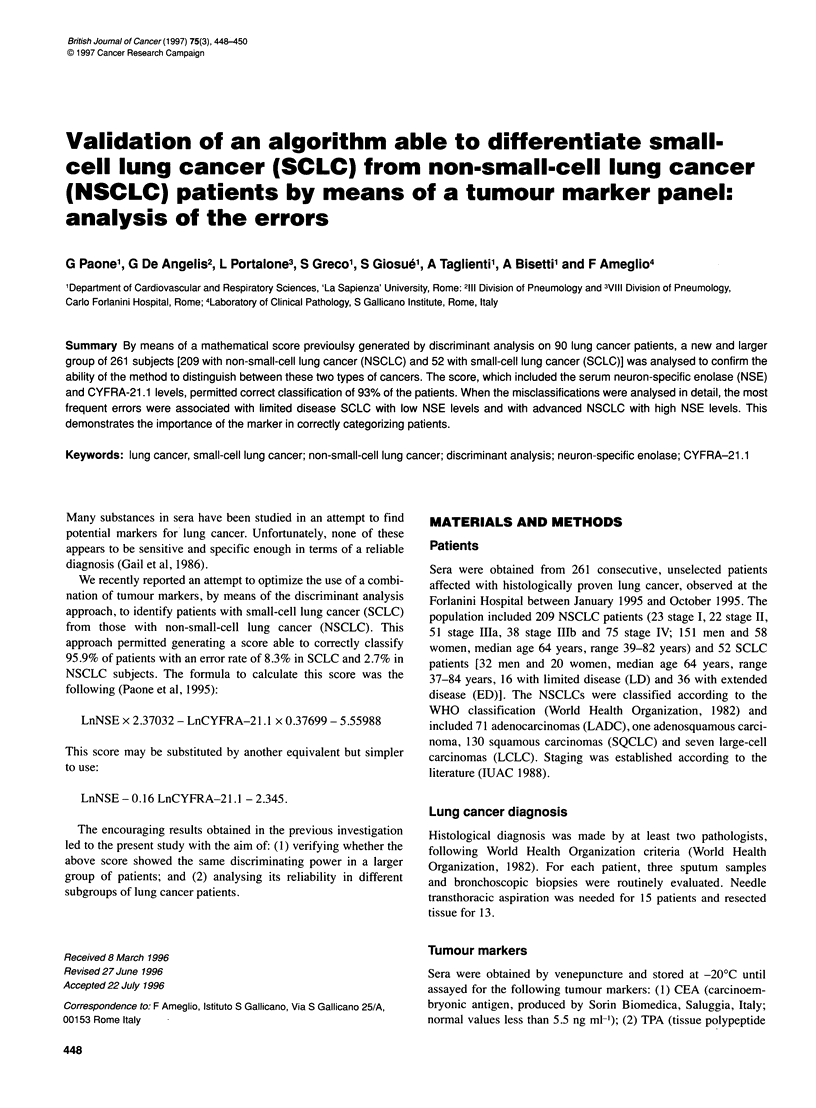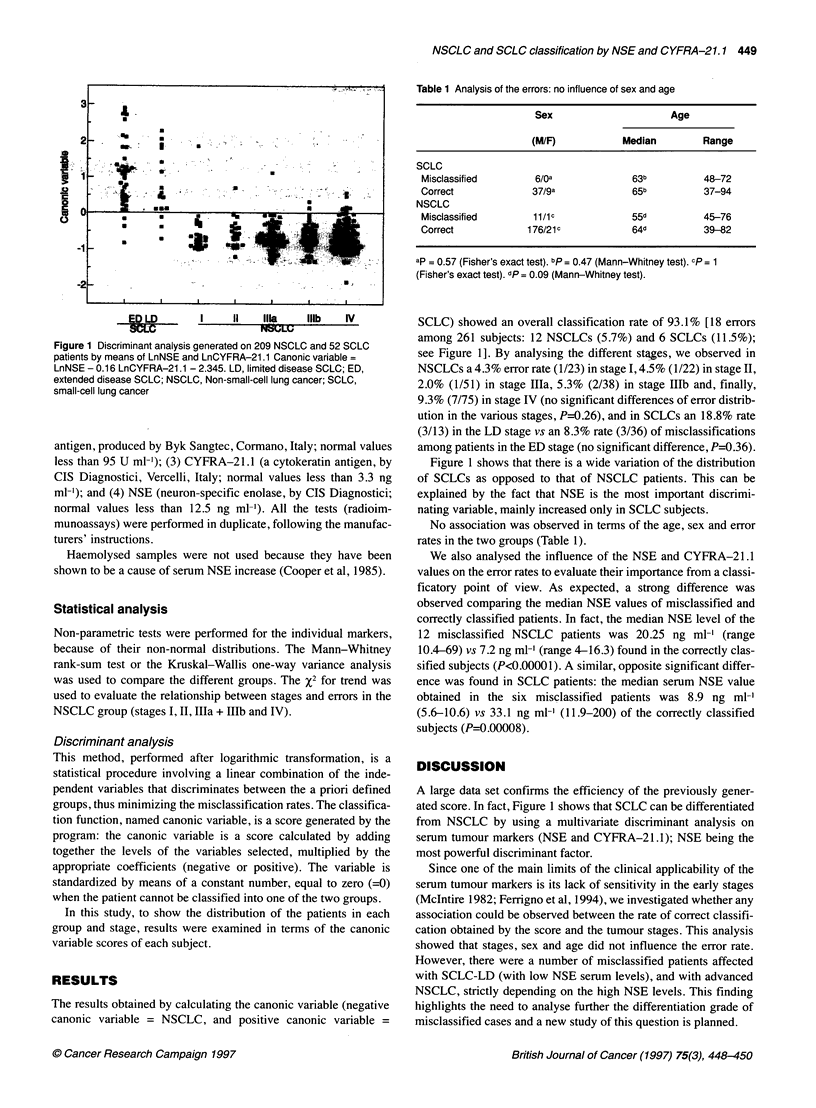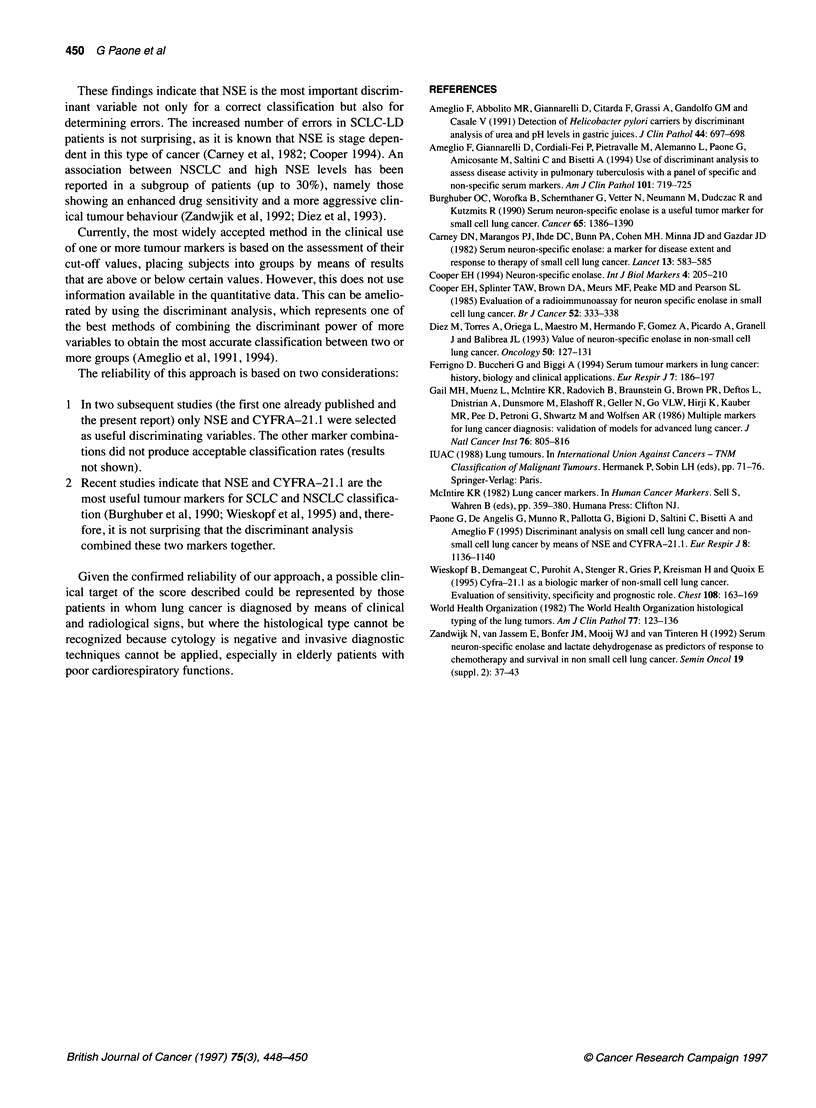# Validation of an algorithm able to differentiate small-cell lung cancer (SCLC) from non-small-cell lung cancer (NSCLC) patients by means of a tumour marker panel: analysis of the errors.

**DOI:** 10.1038/bjc.1997.75

**Published:** 1997

**Authors:** G. Paone, G. De Angelis, L. Portalone, S. Greco, S. Giosué, A. Taglienti, A. Bisetti, F. Ameglio

**Affiliations:** Department of Cardiovascular and Respiratory Sciences, La Sapienza University, Rome, Italy.

## Abstract

By means of a mathematical score previously generated by discriminant analysis on 90 lung cancer patients, a new and larger group of 261 subjects [209 with non-small-cell lung cancer (NSCLC) and 52 with small-cell lung cancer (SCLC)] was analysed to confirm the ability of the method to distinguish between these two types of cancers. The score, which included the serum neuron-specific enolase (NSE) and CYFRA-21.1 levels, permitted correct classification of 93% of the patients. When the misclassifications were analysed in detail, the most frequent errors were associated with limited disease SCLC with low NSE levels and with advanced NSCLC with high NSE levels. This demonstrates the importance of the marker in correctly categorizing patients.


British Journal of Cancer (1997) 75(3), 448–450
© 1997 Cancer Research Campaign

### Validation of an algorithm able to differentiate small-cell lung cancer (SCLC) from non-small-cell lung cancer (NSCLC) patients by means of a tumour marker panel: analysis of the errors

**G Paone[1], G De Angelis[2], L Portalone[3], S Greco[1], S Giosué[1], A Taglienti[1], A Bisetti[1] and F Ameglio[4]**

[1]Department of Cardiovascular and Respiratory Sciences, 'La Sapienza' University, Rome: [2]III Division of Pneumology and [3]VIII Division of Pneumology, Carlo Forlanini Hospital, Rome; [4]Laboratory of Clinical Pathology, S Gallicano Institute, Rome, Italy

**Summary** By means of a mathematical score previoulsy generated by discriminant analysis on 90 lung cancer patients, a new and larger group of 261 subjects [209 with non-small-cell lung cancer (NSCLC) and 52 with small-cell lung cancer (SCLC)] was analysed to confirm the ability of the method to distinguish between these two types of cancers. The score, which included the serum neuron-specific enolase (NSE) and CYFRA-21.1 levels, permitted correct classification of 93% of the patients. When the misclassifications were analysed in detail, the most frequent errors were associated with limited disease SCLC with low NSE levels and with advanced NSCLC with high NSE levels. This demonstrates the importance of the marker in correctly categorizing patients.

**Keywords:** lung cancer, small-cell lung cancer; non-small-cell lung cancer; discriminant analysis; neuron-specific enolase; CYFRA–21.1

Many substances in sera have been studied in an attempt to find potential markers for lung cancer. Unfortunately, none of these appears to be sensitive and specific enough in terms of a reliable diagnosis (Gail et al, 1986).

We recently reported an attempt to optimize the use of a combination of tumour markers, by means of the discriminant analysis approach, to identify patients with small-cell lung cancer (SCLC) from those with non-small-cell lung cancer (NSCLC). This approach permitted generating a score able to correctly classify 95.9% of patients with an error rate of 8.3% in SCLC and 2.7% in NSCLC subjects. The formula to calculate this score was the following (Paone et al, 1995):

$$\text{LnNSE} \times 2.37032 - \text{LnCYFRA–21.1} \times 0.37699 - 5.55988$$

This score may be substituted by another equivalent but simpler to use:

$$\text{LnNSE} - 0.16\ \text{LnCYFRA–21.1} - 2.345.$$

The encouraging results obtained in the previous investigation led to the present study with the aim of: (1) verifying whether the above score showed the same discriminating power in a larger group of patients; and (2) analysing its reliability in different subgroups of lung cancer patients.

*Received 8 March 1996*
*Revised 27 June 1996*
*Accepted 22 July 1996*

*Correspondence to:* F Ameglio, Istituto S Gallicano, Via S Gallicano 25/A, 00153 Rome Italy

### MATERIALS AND METHODS

### Patients

Sera were obtained from 261 consecutive, unselected patients affected with histologically proven lung cancer, observed at the Forlanini Hospital between January 1995 and October 1995. The population included 209 NSCLC patients (23 stage I, 22 stage II, 51 stage IIIa, 38 stage IIIb and 75 stage IV; 151 men and 58 women, median age 64 years, range 39–82 years) and 52 SCLC patients [32 men and 20 women, median age 64 years, range 37–84 years, 16 with limited disease (LD) and 36 with extended disease (ED)]. The NSCLCs were classified according to the WHO classification (World Health Organization, 1982) and included 71 adenocarcinomas (LADC), one adenosquamous carcinoma, 130 squamous carcinomas (SQCLC) and seven large-cell carcinomas (LCLC). Staging was established according to the literature (IUAC 1988).

### Lung cancer diagnosis

Histological diagnosis was made by at least two pathologists, following World Health Organization criteria (World Health Organization, 1982). For each patient, three sputum samples and bronchoscopic biopsies were routinely evaluated. Needle transthoracic aspiration was needed for 15 patients and resected tissue for 13.

### Tumour markers

Sera were obtained by venepuncture and stored at –20°C until assayed for the following tumour markers: (1) CEA (carcinoembryonic antigen, produced by Sorin Biomedica, Saluggia, Italy; normal values less than 5.5 ng ml$^{-1}$); (2) TPA (tissue polypeptide

**Figure 1** Discriminant analysis generated on 209 NSCLC and 52 SCLC patients by means of LnNSE and LnCYFRA-21.1 Canonic variable = LnNSE – 0.16 LnCYFRA-21.1 – 2.345. LD, limited disease SCLC; ED, extended disease SCLC; NSCLC, Non-small-cell lung cancer; SCLC, small-cell lung cancer antigen, produced by Byk Sangtec, Cormano, Italy; normal values less than 95 U ml$^{-1}$); (3) CYFRA-21.1 (a cytokeratin antigen, by CIS Diagnostici, Vercelli, Italy; normal values less than 3.3 ng ml$^{-1}$); and (4) NSE (neuron-specific enolase, by CIS Diagnostici; normal values less than 12.5 ng ml$^{-1}$). All the tests (radioimmunoassays) were performed in duplicate, following the manufacturers' instructions.

Haemolysed samples were not used because they have been shown to be a cause of serum NSE increase (Cooper et al, 1985).

### Statistical analysis

Non-parametric tests were performed for the individual markers, because of their non-normal distributions. The Mann–Whitney rank-sum test or the Kruskal–Wallis one-way variance analysis was used to compare the different groups. The $\chi^2$ for trend was used to evaluate the relationship between stages and errors in the NSCLC group (stages I, II, IIIa + IIIb and IV).

### *Discriminant analysis*

This method, performed after logarithmic transformation, is a statistical procedure involving a linear combination of the independent variables that discriminates between the a priori defined groups, thus minimizing the misclassification rates. The classification function, named canonic variable, is a score generated by the program: the canonic variable is a score calculated by adding together the levels of the variables selected, multiplied by the appropriate coefficients (negative or positive). The variable is standardized by means of a constant number, equal to zero (=0) when the patient cannot be classified into one of the two groups.

In this study, to show the distribution of the patients in each group and stage, results were examined in terms of the canonic variable scores of each subject.

### RESULTS

The results obtained by calculating the canonic variable (negative canonic variable = NSCLC, and positive canonic variable = SCLC) showed an overall classification rate of 93.1% [18 errors among 261 subjects: 12 NSCLCs (5.7%) and 6 SCLCs (11.5%); see Figure 1]. By analysing the different stages, we observed in NSCLCs a 4.3% error rate (1/23) in stage I, 4.5% (1/22) in stage II, 2.0% (1/51) in stage IIIa, 5.3% (2/38) in stage IIIb and, finally, 9.3% (7/75) in stage IV (no significant differences of error distribution in the various stages, $P$=0.26), and in SCLCs an 18.8% rate (3/13) in the LD stage *vs* an 8.3% rate (3/36) of misclassifications among patients in the ED stage (no significant difference, $P$=0.36).

**Table 1** Analysis of the errors: no influence of sex and age

| | Sex | Age | |
|---|---|---|---|
| | (M/F) | Median | Range |
| SCLC | | | |
| Misclassified | 6/0[a] | 63[b] | 48–72 |
| Correct | 37/9[a] | 65[b] | 37–94 |
| NSCLC | | | |
| Misclassified | 11/1[c] | 55[d] | 45–76 |
| Correct | 176/21[c] | 64[d] | 39–82 |

[a]P = 0.57 (Fisher's exact test). [b]$P$ = 0.47 (Mann–Whitney test). [c]$P$ = 1 (Fisher's exact test). [d]$P$ = 0.09 (Mann–Whitney test).

Figure 1 shows that there is a wide variation of the distribution of SCLCs as opposed to that of NSCLC patients. This can be explained by the fact that NSE is the most important discriminating variable, mainly increased only in SCLC subjects.

No association was observed in terms of the age, sex and error rates in the two groups (Table 1).

We also analysed the influence of the NSE and CYFRA-21.1 values on the error rates to evaluate their importance from a classificatory point of view. As expected, a strong difference was observed comparing the median NSE values of misclassified and correctly classified patients. In fact, the median NSE level of the 12 misclassified NSCLC patients was 20.25 ng ml$^{-1}$ (range 10.4–69) *vs* 7.2 ng ml$^{-1}$ (range 4–16.3) found in the correctly classified subjects ($P$<0.00001). A similar, opposite significant difference was found in SCLC patients: the median serum NSE value obtained in the six misclassified patients was 8.9 ng ml$^{-1}$ (5.6–10.6) *vs* 33.1 ng ml$^{-1}$ (11.9–200) of the correctly classified subjects ($P$=0.00008).

### DISCUSSION

A large data set confirms the efficiency of the previously generated score. In fact, Figure 1 shows that SCLC can be differentiated from NSCLC by using a multivariate discriminant analysis on serum tumour markers (NSE and CYFRA-21.1); NSE being the most powerful discriminant factor.

Since one of the main limits of the clinical applicability of the serum tumour markers is its lack of sensitivity in the early stages (McIntire 1982; Ferrigno et al, 1994), we investigated whether any association could be observed between the rate of correct classification obtained by the score and the tumour stages. This analysis showed that stages, sex and age did not influence the error rate. However, there were a number of misclassified patients affected with SCLC-LD (with low NSE serum levels), and with advanced NSCLC, strictly depending on the high NSE levels. This finding highlights the need to analyse further the differentiation grade of misclassified cases and a new study of this question is planned.

© Cancer Research Campaign 1997 

These findings indicate that NSE is the most important discriminant variable not only for a correct classification but also for determining errors. The increased number of errors in SCLC-LD patients is not surprising, as it is known that NSE is stage dependent in this type of cancer (Carney et al, 1982; Cooper 1994). An association between NSCLC and high NSE levels has been reported in a subgroup of patients (up to 30%), namely those showing an enhanced drug sensitivity and a more aggressive clinical tumour behaviour (Zandwjik et al, 1992; Diez et al, 1993).

Currently, the most widely accepted method in the clinical use of one or more tumour markers is based on the assessment of their cut-off values, placing subjects into groups by means of results that are above or below certain values. However, this does not use information available in the quantitative data. This can be ameliorated by using the discriminant analysis, which represents one of the best methods of combining the discriminant power of more variables to obtain the most accurate classification between two or more groups (Ameglio et al, 1991, 1994).

The reliability of this approach is based on two considerations:

1 In two subsequent studies (the first one already published and the present report) only NSE and CYFRA–21.1 were selected as useful discriminating variables. The other marker combinations did not produce acceptable classification rates (results not shown).
2 Recent studies indicate that NSE and CYFRA–21.1 are the most useful tumour markers for SCLC and NSCLC classification (Burghuber et al, 1990; Wieskopf et al, 1995) and, therefore, it is not surprising that the discriminant analysis combined these two markers together.

Given the confirmed reliability of our approach, a possible clinical target of the score described could be represented by those patients in whom lung cancer is diagnosed by means of clinical and radiological signs, but where the histological type cannot be recognized because cytology is negative and invasive diagnostic techniques cannot be applied, especially in elderly patients with poor cardiorespiratory functions.

### REFERENCES

Ameglio F, Abbolito MR, Giannarelli D, Citarda F, Grassi A, Gandolfo GM and Casale V (1991) Detection of *Helicobacter pylori* carriers by discriminant analysis of urea and pH levels in gastric juices. *J Clin Pathol* **44**: 697–698

Ameglio F, Giannarelli D, Cordiali-Fei P, Pietravalle M, Alemanno L, Paone G, Amicosante M, Saltini C and Bisetti A (1994) Use of discriminant analysis to assess disease activity in pulmonary tuberculosis with a panel of specific and non-specific serum markers. *Am J Clin Pathol* **101**: 719–725

Burghuber OC, Worofka B, Schernthaner G, Vetter N, Neumann M, Dudczac R and Kutzmits R (1990) Serum neuron-specific enolase is a useful tumor marker for small cell lung cancer. *Cancer* **65**: 1386–1390

Carney DN, Marangos PJ, Ihde DC, Bunn PA, Cohen MH. Minna JD and Gazdar JD (1982) Serum neuron-specific enolase: a marker for disease extent and response to therapy of small cell lung cancer. *Lancet* **13**: 583–585

Cooper EH (1994) Neuron-specific enolase. *Int J Biol Markers* **4**: 205–210

Cooper EH, Splinter TAW, Brown DA, Meurs MF, Peake MD and Pearson SL (1985) Evaluation of a radioimmunoassay for neuron specific enolase in small cell lung cancer. *Br J Cancer* **52**: 333–338

Diez M, Torres A, Oriega L, Maestro M, Hermando F, Gomez A, Picardo A, Granell J and Balibrea JL (1993) Value of neuron-specific enolase in non-small cell lung cancer. *Oncology* **50**: 127–131

Ferrigno D. Buccheri G and Biggi A (1994) Serum tumour markers in lung cancer: history, biology and clinical applications. *Eur Respir J* **7**: 186–197

Gail MH, Muenz L, McIntire KR, Radovich B, Braunstein G, Brown PR, Deftos L, Dnistrian A, Dunsmore M, Elashoff R, Geller N, Go VLW, Hirji K, Kauber MR, Pee D, Petroni G, Shwartz M and Wolfsen AR (1986) Multiple markers for lung cancer diagnosis: validation of models for advanced lung cancer. *J Natl Cancer Inst* **76**: 805–816

IUAC (1988) Lung tumours. In *International Union Against Cancers – TNM Classification of Malignant Tumours*. Hermanek P, Sobin LH (eds), pp. 71–76. Springer-Verlag: Paris.

McIntire KR (1982) Lung cancer markers. In *Human Cancer Markers*. Sell S, Wahren B (eds), pp. 359–380. Humana Press: Clifton NJ.

Paone G, De Angelis G, Munno R, Pallotta G, Bigioni D, Saltini C, Bisetti A and Ameglio F (1995) Discriminant analysis on small cell lung cancer and non-small cell lung cancer by means of NSE and CYFRA–21.1. *Eur Respir J* **8**: 1136–1140

Wieskopf B, Demangeat C, Purohit A, Stenger R, Gries P, Kreisman H and Quoix E (1995) Cyfra–21.1 as a biologic marker of non-small cell lung cancer. Evaluation of sensitivity, specificity and prognostic role. *Chest* **108**: 163–169

World Health Organization (1982) The World Health Organization histological typing of the lung tumors. *Am J Clin Pathol* **77**: 123–136

Zandwijk N, van Jassem E, Bonfer JM, Mooij WJ and van Tinteren H (1992) Serum neuron-specific enolase and lactate dehydrogenase as predictors of response to chemotherapy and survival in non small cell lung cancer. *Semin Oncol* **19** (suppl. 2): 37–43

© Cancer Research Campaign 1997